# Are slum dwellers at heightened risk of HIV infection than other urban residents? Evidence from population-based HIV prevalence surveys in Kenya

**DOI:** 10.1016/j.healthplace.2012.04.003

**Published:** 2012-09

**Authors:** Nyovani J. Madise, Abdhalah K. Ziraba, Joseph Inungu, Samoel A. Khamadi, Alex Ezeh, Eliya M. Zulu, John Kebaso, Vincent Okoth, Matilu Mwau

**Affiliations:** a University of Southampton, Division of Social Statistics and Centre for Global Health, Population, Poverty, and Policy, University of Southampton, Southampton SO17 1BJ, United Kingdom; b Faculty of Epidemiology and Population Health, Department for Population Studies, London School of Hygiene and Tropical Medicine, Keppel Street, London WC1E 7HT, United Kingdom; c Population Services International, 08 BP 0876 Tri Postal, Cotonou, Benin; d Kenya Medical Research Institute, P.O. Box 54840-00200, Nairobi, Kenya; e African Population and Health Research Center, P.O. Box 10787, 00100 GPO, Nairobi, Kenya; f African Institute for Development Policy (AFIDEP), P.O. Box 14688-00800, Nairobi, Kenya; g Loma Linda University, School of Public Health, 24951 North Circle Drive, Nichol Hall 1410, Loma Linda, CA 92350, USA

**Keywords:** HIV prevalence, Intra-urban, Slums, Kenya

## Abstract

In 2008, the global urban population surpassed the rural population and by 2050 more than 6 billion will be living in urban centres. A growing body of research has reported on poor health outcomes among the urban poor but not much is known about HIV prevalence among this group. A survey of nearly 3000 men and women was conducted in two Nairobi slums in Kenya between 2006 and 2007, where respondents were tested for HIV status. In addition, data from the 2008/2009 Kenya Demographic and Health Survey were used to compare HIV prevalence between slum residents and those living in other urban and rural areas. The results showed strong intra-urban differences. HIV was 12% among slum residents compared with 5% and 6% among non-slum urban and rural residents, respectively. Generally, men had lower HIV prevalence than women although in the slums the gap was narrower. Among women, sexual experience before the age of 15 compared with after 19 years was associated with 62% higher odds of being HIV positive. There was ethnic variation in patterns of HIV infection although the effect depended on the current place of residence.

## Highlights

► HIV prevalence in Nairobi slums is 12%, compared with 5% among non-slum urban residents, and 6% in rural areas. ► HIV prevalence in Kenyan urban areas is principally fuelled by very high HIV infection rates in slum areas. ► Women who have first sexual intercourse at early ages are at increased risk of becoming HIV infected. ► In Kenya there are strong patterns of HIV infection by ethnicity but living in urban areas dilutes this effect.

## 1 Introduction

The HIV epidemic has proved to be the biggest global public health challenge of the 21st Century. More than 30 million people were living with HIV in 2010 with 2.6 million people newly infected that year alone (UNAIDS, 2010). While sub-Saharan Africa is home to only 12.5% of the world's population, it accounts for more than 67% of the world's HIV-infected people and about 90% of the 2 million children under the age of 15 years who are infected with the HIV virus (UNAIDS, 2010). The availability of population-based HIV data such as those collected under the Demographic and Health Surveys (DHS) programme has made it possible to track the epidemic and to identify sub-groups most at risk of HIV infection. However, getting accurate estimates for sub-populations such as the urban poor is often hampered by lack of data since many DHS samples do not contain large enough samples to tease out intra-urban differences. The focus on the urban poor is warranted since globally, there are now more people living in urban areas than in rural areas and the United Nations projections are that the urban population will increase by 2.5 billion to 6.3 billion by 2050. Virtually all of this urban population growth will be in the urban areas of less developed regions, especially in Africa and Asia (United Nations Population Division, 2007).

**Fig. 1:**
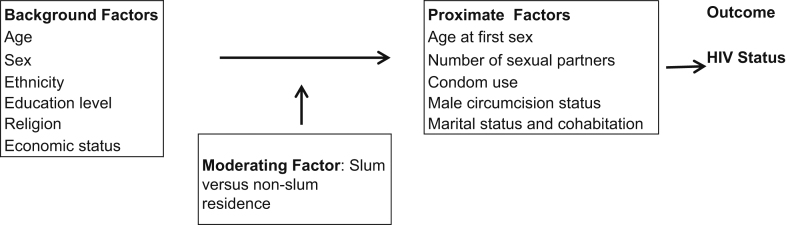
Conceptual framework of the relationship between HIV status and slum residence.

In recent years evidence has grown of large health inequalities between the urban poor and other residents (Bocquier et al., 2011; Fotso et al., 2007; Harpham, 2009; Satterthwaite, 1995). Much of this disadvantage is attributed to poor environmental conditions and lack of adequate sanitation in slum areas where most urban poor people live (United Nations Human Settlements Programme, 2003). In virtually all of sub-Saharan Africa, urban residents have higher HIV prevalence than rural residents, but there is limited knowledge of the HIV infection rates of different socio-economic groups within urban areas. Ample evidence exists though of risky sexual practices among slum residents. These include early age at sexual debut, multiple sexual partners, transactional sex, and low condom use (Adedimeji et al., 2007; Dodoo et al., 2002; Zulu et al., 2002). It is thought that these risky sexual practices are common because of economic hardships, overcrowding, lack of security, separate spousal living arrangements, and early socialisation of children into sex due to lack of privacy and exposure to pornographic material (Greif et al., 2011; Greif and Nii-Amoo Dodoo, 2011; Mbirimtengerenji, 2007; Zulu et al., 2003). Conceptually, slum residence can be viewed as a moderating variable, which intensifies or attenuates the effects of background characteristics such as current age, sex, economic status, and education level to influence the proximate determinants of HIV status (see Fig. 1).

Our study was the first to document the high HIV prevalence in slum settings using a population-based HIV survey (Ziraba et al., 2010). In this paper, we use data on HIV status collected in two slum areas in Nairobi and the 2008/2009 Kenya DHS to compare patterns of HIV infection by place of residence (slum, non-slum urban, and rural) while controlling for demographic and socio-economic factors.

## 2 Background

Nairobi city, like many other cities in the developing world, has experienced rapid population growth over the last four decades following independence. In 1948, there were an estimated 120,000 living in the city and according to the 2009 Kenya Population and Housing Census, the city had grown to 3.2 million inhabitants (Kenya National Bureau of Statistics (KNBS), 2010). An estimated 60% of the urban population are thought to be living in slums (United Nations Population Division, 2008). The housing structures in the slums are temporary with no proper planning, and there is overcrowding, poor sanitation, and insecurity (African Population and Health Research Center, 2002). Unemployment is high, and the majority of residents are involved in petty trading or casual labour (Beguy et al., 2010). Given that these settlements are considered illegal, there is some reluctance by the authorities to provide services such as schools, roads, and healthcare facilities. As a consequence, residents of these settlements have higher mortality and low healthcare utilisation compared with other urban residents (Beguy et al., 2010; Kyobutungi et al., 2007; Ziraba et al., 2009a, 2009b).

About 6% of Kenyan adults are thought to be HIV positive (Kenya National Bureau of Statistics and ICF Macro, 2010). Prior to our study, little was known about the HIV infection among the urban poor even though HIV/AIDS was acknowledged to contribute to high mortality among slums dwellers. Two studies using verbal autopsy data from the two Nairobi slums showed that more than 50% of the adult mortality burden and late maternal deaths could be attributable to HIV/AIDS and tuberculosis (Kyobutungi et al., 2007; Ziraba et al., 2009a). However, HIV status was not established by serological testing in these studies.

## 3 Data and methods

The Nairobi Slum HIV Prevalence Survey (NSHPS) was conducted collaboratively by the African Population and Health Research Center (APHRC) and Kenya Medical Research Institute (KEMRI) between 2006 and 2007. The aim was to estimate HIV prevalence in two slums in Nairobi city, namely, Korogocho and Viwandani, where APHRC runs the Nairobi Urban Health and Demographic Surveillance System (NUHDSS). The NUHDSS is a longitudinal surveillance of about 60,000 individuals who are visited once every 4 months. Ethical clearance for HIV testing was obtained from the Kenya Medical Research Institute Ethical Review Committee. Simple random sampling with the aid of computer generated random numbers was used to select men and women from a pre-stratified list from the NUHDSS database. Participants had to be residents of the two slums, registered by the NUHDSS, aged 15–49 years for females or 15–54 years for males, and able to participate in the interview. Participants were given unique HIV study identifiers distinct from the NUHDSS identifiers to maintain confidentiality. Only the principal investigator and the senior data analyst had the key to match the two identifiers.

Before field work began, community meetings were held to explain the purpose of the study. In addition, every participant was given information about the objectives of the study and informed consent was affirmed by signing a consent form. The interviewer read out the informed consent to those who could not read. Minors (15–17 years old) who agreed to participate signed the informed consent and in addition, their guardians also provided consent by appending their signature or thumb prints. The survey used face-to-face interviewing to collect data on knowledge of HIV prevention, HIV testing history, marriage and sexual activity, and circumcision. HIV status was determined serologically using the Determine HIV-1/HIV-2 (Abbott) and Uni-Gold™ rapid test kits.

Initially, a pilot was conducted with a simple random sample of 800 participants in the two slums. The size of the pilot was deliberately large to be able to estimate HIV prevalence in each of the two slum communities. Following the successful pilot, the sample was increased to 5004 to enable estimation of HIV prevalence by demographic and socio-economic characteristics. About 76% of women who were sampled were contacted while the contact rate for men was 70%. With the benefit of NUHDSS updates on residency status of individuals under surveillance, 237 in the original sample were identified as not eligible residents (i.e. had not lived in the area for the required 120 days according to the NUHDSS rules). Thus, the number of people who were eligible from the initial sample was 4767, of which 61% were female. Out of these, 2590 participated in both the survey and agreed to be tested, 131 gave blood samples only, and 573 accepted to be interviewed only. About 30% of those eligible refused to participate or were not found at home after three visits to their households.

We assessed the likely effect of non-participation on the HIV prevalence estimates and found that overall, the prevalence of HIV could have been marginally overestimated by 2% with underestimation among men of 1% and overestimation among women of 3%. However, all the corrected estimates lie within the confidence limits of the unadjusted prevalence estimate (Ziraba et al., 2010). Based on this finding, in this paper we do not correct the data for non-response.

We used data from the 2008/2009 Kenya DHS to derive the comparator sub-populations of rural and non-slum urban residents. Since the DHS might have sampled residents of slums areas, we sought to identify such households. The UN Habitat definition of an urban slum household is one that lacks any of five elements: access to improved water; access to improved sanitation; security of tenure; durability of housing; and sufficient living area (UN Habitat, 2010). A strict application of these criteria leads to a large and rather heterogeneous slum community and most scholars have refined this definition of a slum as “lacking two or more of the elements in the UN Habitat list” (Fotso et al., 2007; Zulu et al., 2002). In this paper, we used the method proposed by Zulu et al. (2002), who defined an urban slum household as one that had no flush toilet, no piped water nor electricity. From the DHS, 21 residents in Nairobi city and 306 from other urban areas outside Nairobi city (mainly Mombasa and Kisumu) were identified as living in slums by this method.

We examined HIV status by background demographic and socioeconomic characteristics namely: current age, age at first sex, ethnicity, marital status, highest level of education attained, and wealth status. Wealth status was determined by constructing quintiles based on Filmer and Pritchett's principal component analysis methodology (Filmer and Pritchett, 2001). Quintiles were derived separately for all rural, urban, and the slum areas. For men we also examined HIV prevalence by circumcision status but this variable was not included in the multivariate because it was of borderline insignificance. The 2008/2009 Kenya DHS is not self-weighting since urban areas were over-sampled to obtain enough cases for analysis (Kenya National Bureau of Statistics and ICF Macro, 2010). We therefore used weighted data to derive estimates of HIV prevalence but used unweighted data for the multivariate analyses. For the multivariate analysis, we pooled the data so that we could estimate the odds of being HIV positive by place of residence. Separate logistic regression models were conducted for men and women since preliminary analyses pointed to many statistically significant interactions involving the sex of the respondent, suggesting that separate analyses were appropriate. Since place of residence is viewed as a moderating variable in this paper, we tested interactions between place of residence and the background factors (for example age, educational level, ethnicity, and wealth status). Among the proximate factors highlighted in our conceptual framework (Fig. 1) we tested the significance of the age at first sex, male circumcision, and marital status. We did not include condom use and number of sexual partners because of the potential reverse causality. The results of the multivariate analysis are presented as odds ratios and where there are significant interactions, estimated probabilities are used to explain the interaction effects.

## 4 Results

Table 1 shows the percentage distribution of all respondents in the 2006/2007 NSHPS and 2008/2009 Kenya DHS by place of residence and selected background characteristics. About 29% of the respondents in the pooled dataset were from Nairobi slums, 3.2% were from other slums, 8.2% from other urban non-slum areas, and 54% were from rural areas. The age composition of the respondents showed higher proportions in the age ranges 20–29 among urban residents compared with the rural population. By design, the NSHPS sampled more women than men, which is reflected in there being more women from Nairobi slums than men. In terms of education, the slum population was similar to the rural population with the majority having received primary education only. In contrast, about 73% of respondents in non-slum Nairobi had attained secondary or higher education and in other non-slum areas the corresponding percentage was 57. With respect to the ethnic composition, about 85% of slum residents belonged to the four main tribes (Kikuyu, Kamba, Luhya, and Luo), while the non-Nairobi population was more diverse.

Since the sample size of “other slums” was small (*n*=306) and because there was no significant difference in HIV prevalence between this group and the Nairobi slum sub-population, the two groups were combined for subsequent analyses. Similarly, the non-slum urban group from other parts of the country was combined with the Nairobi non-slum urban group.

**Fig. 2:**
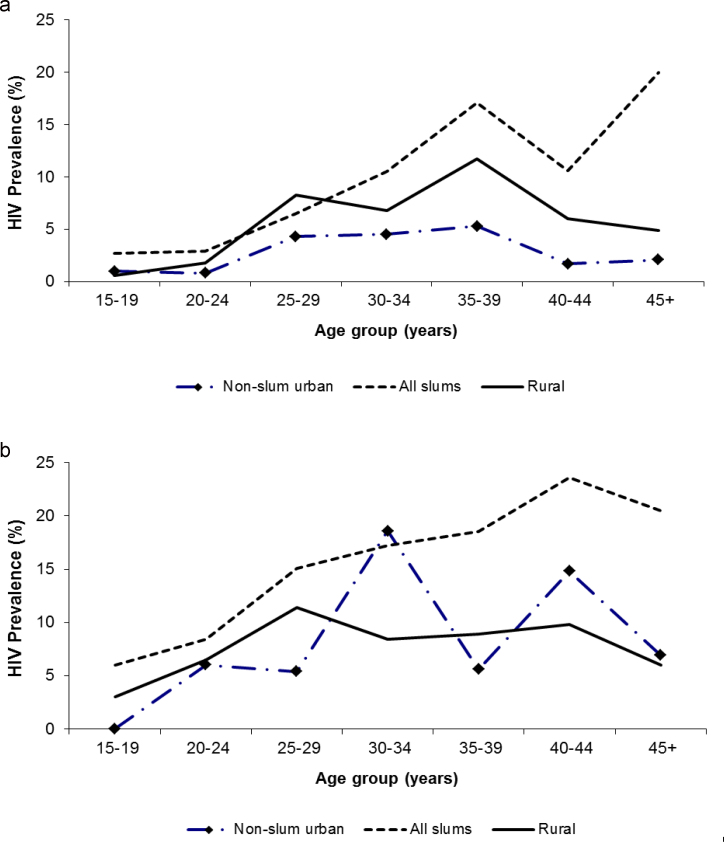
HIV prevalence among men and women by age and place: 2006/2007 Nairobi Slum HIV prevalence study and 2008/2009 Kenya DHS.

Table 2 shows the HIV prevalence by selected background characteristics. Overall, about 12% of slum residents were HIV positive, compared with 5% of non-slum urban and 6% of rural residents. Generally, men had lower HIV prevalence than women as expected, but the gap in slum areas was narrower. Women in slums had 38% higher HIV prevalence than men while the corresponding excess risks for rural and non-slum urban areas were 60% and 166%, respectively. The age patterns of HIV infection varied by sex and the place of residence. This is illustrated further in Fig. 2a and b. Taking the men's distribution (Fig. 2a), the HIV prevalence among men who were resident in the slums steadily increased with age, with a dip at 40–44 years. For rural men, HIV prevalence declined with age after 35 years. Apart from the 15–19 years age-group, non-slum urban men had lower HIV prevalence across all age-groups compared with men residing in other places. The pattern for women (Fig. 2b) showed higher HIV prevalence among slum residents as expected. However, unlike the pattern for men, women who were living in non-slum urban areas did not have lower HIV prevalence generally. In particular, those in the age ranges of 30–34 years and 40–44 years had much higher HIV prevalence compared with rural women of similar ages.

From Table 2, we note that there were large differences in the HIV prevalence rates between the different ethnic groups. HIV was the highest among the Luo tribe as noted in all population-based HIV studies in Kenya. Those who had never been married nor been in a formal sexual union had the lowest HIV rates and those who had been previously married had the highest rates. Early age at first sexual intercourse was associated with higher HIV rates especially among women. For example, HIV prevalence was about 25% among slum and non-slum urban women who had first sexual intercourse before the age of 15 years. In contrast, only about 5% of women in non-slum urban areas and 13% of slum women who had first sexual intercourse after 20 years were HIV positive. The protective effect of circumcision for HIV among men was more pronounced in slum and rural areas and less so in non-slum urban areas.

Table 3 shows the results of the logistic regression analysis for the pooled data, analysed separately for men and women. The first panel shows the odds ratios of being HIV positive by place of residence, unadjusted for other background variables and the second panel shows the adjusted odds ratios. We tested for interactions in the multivariable logistic regression model and only one for the women's model was significant, between the place of residence and ethnicity (*p*-value=0.012). An interaction between male circumcision and place of residence for the men's model was of borderline significance (*p*-value=0.05). This showed lower likelihood of being HIV positive in rural and slum areas among circumcised men compared with those who were uncircumcised. However, because the significance was borderline, and to reduce complexity of the models, this interaction is not shown in Table 3. No other interactions were statistically significant at 5% level.

Turning to the first panel showing unadjusted odds ratios, men who lived in non-slum urban areas had 0.39 times the odds of being HIV positive compared with men in slum areas. The corresponding odds ratio of being HIV positive for rural men compared with those living in slums was 0.44. Among women, the effect was weaker, but still statistically significant. Compared with women living in slums, the odds ratio of being HIV positive for non-slum urban women was 0.61 and for rural women it was 0.52. After adjusting for age, ethnicity, marital status, age at first sex, education attainment, and wealth status, the odds of being HIV positive for men in slums were still significantly higher than those of men in non-slum urban areas (OR=0.51) and in rural areas (OR=0.61). For women, there was a significant interaction between place of residence and ethnicity which is described fully below. With respect to age, there was no significant difference in the odds of being HIV positive between men in the age ranges of 15–24 years. Among older men, the odds of being HIV positive were between 3 and 7.5 times higher compared with those of 15–19 years. Men who were 35–39 years old had the highest odds of being HIV positive (OR=7.5). For women, there was no significant difference in the odds of being HIV positive for women aged 15–19 compared with those 20–24 years or those 45 years or older. The odds of being HIV positive among those in the age ranges of 25–44 years were between 2.3 and 2.7 times higher than the odds for women 15–19 years old.

Men and women who had never been married had lower odds of being HIV positive compared with those who were divorced or widowed (OR=3.6 for men and OR=3.8 for women). There was no statistically significant difference in the likelihood of being HIV positive between those who had never married and those who were in stable sexual unions at the time of the survey. The age at first sex was statistically significant only in the women's model and showed that those who had sex before the age of 15 years had much higher odds of being HIV positive than those who became sexually active at later ages. In particular, having first sex after the age of 20 years was associated with a 62% reduction in the odds of being HIV positive compared with being sexually active before the age of 15.

**Fig. 3:**
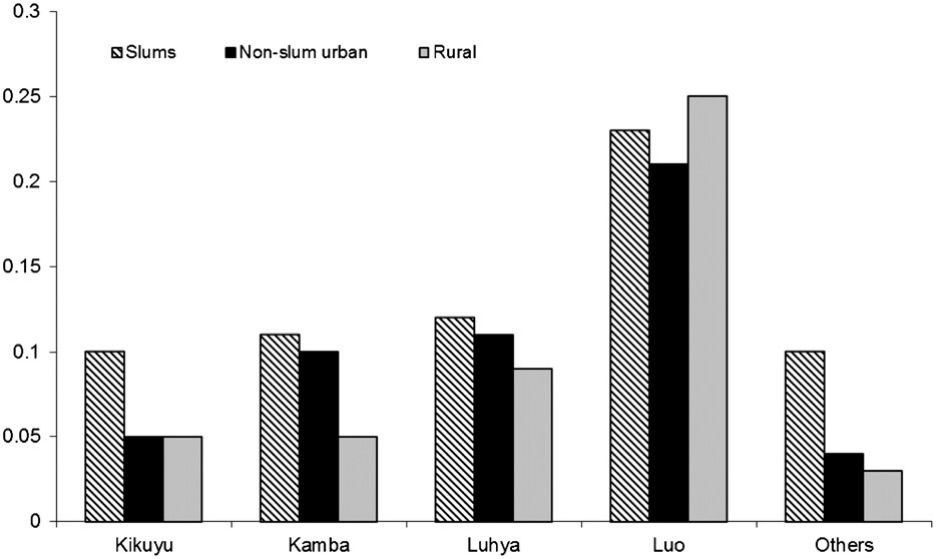
Estimated probabilities of being HIV positive among women by place of residence and ethnicity: 2006/2007 Nairobi Slum HIV Prevalence Survey and 2008/2009 Kenya DHS.

In Kenya, ethnic variation in HIV prevalence is well documented. The findings from this study supported the well-known fact of high HIV prevalence among the Luos, followed by Luhya tribes. An interesting finding was the significant interaction between ethnicity and place of residence in the women's model. Generally, HIV prevalence was higher in slum than non-slum areas but some patterns emerged. Among the Luo women, the highest probability of being HIV positive was among those in rural areas (0.25), followed by those in slums (0.23), and non-slum urban women (0.21)—see Fig. 3. Among the other tribes, the pattern showed higher likelihood of being HIV positive in slums, followed by non-slum urban areas and lowest in rural areas, except that among Kambas and Luhyas, there was not much difference between slum and non-slum urban dwellers.

The socio-economic variables (educational attainment and wealth status) were not significantly associated with the odds of being HIV positive after the other variables were included in the models. We analysed the slum data alone to see the relative importance of the socio-economic variables among this group and found that educational attainment was not significant for both men's and women's models. Wealth status was of borderline significance in the women's model only (p=0.05) and showed slightly lower odds of HIV infection among the 40% least poor.

## 5 Discussion

In most of sub-Saharan Africa, HIV prevalence is typically higher in urban than in rural settings and Kenya is no different. There is ample literature that shows that residents of slum areas engage in riskier sexual practices than other sub-groups but studies that demonstrate the effect on HIV prevalence or incidence have so far been lacking. Our study of HIV prevalence in the slums of Nairobi city of Kenya was the first to confirm that strong intra-urban differences in the risk of HIV infection exist. In Kenya, the higher urban HIV prevalence appears to be principally driven by high rates of HIV infection in slum areas. Earlier studies conducted in Kenyan slums have reported of early initiation of sex, high prevalence of multiple sexual partnerships, and low use of condoms among residents of slums compared with non-slum urban and rural residents (Dodoo et al., 2002; Zulu et al., 2002, 2003). Thus, it is not surprising that slum areas have high HIV infection rates.

The relationship between HIV infection and age is universally acknowledged since age is strongly linked to sexual experience, frequency of sex, and risk-taking. The age pattern of HIV infection in most African populations has been an inverted U-shape, with prevalence among those under 20 years and those over 40 years significantly lower than those in the middle years (Montana et al., 2007). For the slum population however, we do not see the decline in HIV prevalence rates after the age of 40 years except for a dip between the ages of 40 and 44 years among men. The overall HIV pattern among slum residents is of increasing HIV rates with age. One hypothesis of this age pattern is that long periods of exposure to the slum environment could be increasing the risk of HIV infection among older residents. Because most migrants into slums are under the age of 25 years, a large percentage of older slum residents may be those who have lived in the slums for a long period of time (Beguy et al., 2010). To test this hypothesis, we would require detailed migration histories and longitudinal data on HIV infection which are beyond the scope of this paper. Another interpretation of the increasing HIV rates by age is that the multiplicity of high risk sexual activity and its drivers seem to work their way even to older ages.

Ethnic variations in HIV are often taken to signify cultural differences in practices that are associated with HIV infection. In Kenya, the Luo ethnic group has the highest HIV rates. Many reasons have been put forward to explain this, for example lower rates of male circumcision, widow inheritance, and widow cleansing (a practice where a widow engages in sexual intercourse during funeral rites as a way of purification; Akwara et al., 2003; Bailey et al., 2002). There are also reports that Luos have higher exposure to blood contamination because of tattooing and body piercing by traditional healers (Ounga et al., 2009). Our study confirms the high HIV prevalence among Luos even in urban areas. An interesting finding from this study is that among women, ethnic patterns appear to be confounded by the current place of residence. Living in urban areas appears to alter the risks of infection associated with a particular ethnic group. One reason for this is that people living in urban areas may be less likely to adhere to cultural practices that increase or decrease the risk of HIV infection. Furthermore, there are higher chances of intermarrying between ethnic groups in urban areas, which could lead to dilution of customary practices.

The literature on marriage and HIV infection in Africa is rather mixed. Some authors have found that early marriage is a risk factor for women chiefly because of the age difference between spouses, increased coital frequency, and lack of condom use (Clark, 2004) and others have reported that late marriage is associated with higher HIV prevalence probably because of long periods of exposure to premarital sex and frequent partner exchanges (Bongaarts, 2007). In our study, there is no difference in HIV rates between unmarried and currently married men and women. However, marital dissolution either through divorce or widowhood is associated with elevated risks of HIV infection. In part this is explained by the fact that in high HIV prevalent countries such as Kenya, a disproportionate number of dead spouses are HIV infected and their spouses may be infected also. Among divorcees, the situation may be more complex. Reniers has proposed hypotheses that men and women use marriage (or its dissolution) as a strategy for regulating exposure to HIV infection (Reniers, 2008). He argues that men or women may divorce an unfaithful spouse to minimise their risks of infection although he admits that this strategy may be more open to men than women. He also hypothesises that there may be positive selection among the pool of divorcees, whereby those who are HIV negative are more likely to remarry than those who are HIV positive. These hypotheses broadly concur with our findings but detailed studies of marriage and remarriage in the context of HIV are needed to come up with firmer conclusions.

Surprisingly, socioeconomic factors such as educational attainment and wealth status were not significantly associated with the risk of being HIV positive. A positive association between wealth and HIV status has been reported in a number of studies using cross-country comparisons (Mishra et al., 2007; Parkhurst, 2010; Shelton et al., 2005). One argument for a positive association between wealth and HIV status reported in early studies is that at the early stages of the epidemic, wealthier people have higher rates of partner change because they can afford to pay for sex and they are more mobile (Hargreaves and Howe, 2010). As the epidemic progresses and rates of HIV infection become higher, the distinction between the socio-economic classes decreases. Indeed, Gillespie et al. (2007) argue that the association between wealth and HIV status at a national level may be spurious since urban residents tend to be richer than rural residents, and urban HIV rates are higher and that once urban/rural residence is controlled for, the association disappears. Other authors have found an inconsistent association between wealth and HIV status, leading to conclusions that the context and stage of the epidemic matters (Booysen, 2004).

## 6 Conclusions

This study has highlighted the importance of acknowledging intra-urban differences when studying HIV patterns. In this paper, we found that high urban HIV prevalence in Kenya was largely driven by very high prevalence among slum dwellers. Slum dwellers in Kenya appear to be at heightened risk of HIV infection although this effect is slightly attenuated when age, ethnicity, and age at sexual debut are controlled for. Among slum residents, the risks of being infected by HIV remain high even among older residents. We recommend that population-based HIV surveys should include older people to help us understand the HIV situation at older ages. The study also highlighted the vulnerability of women to HIV infection especially when they become sexually experienced at very young ages. Addressing risky sexual practices such as early sexual debut is one strategy which could lead to lower HIV rates among slum dwellers. In addition, addressing the lack of security and sexual violence in the slums could confer protection among young girls and women. Contrary to what has been published in many reports, we found that socio-economic status was not positively associated with HIV status in these samples but found that current age, marital status, and ethnicity were the most important in explaining the differences in HIV rates. Overall, we recommend that HIV prevention efforts and treatment programmes should target the urban poor and they should acknowledge that city dwellers are not a homogeneous group.

## Figures and Tables

**Table 1:** Percentage distribution of respondents by selected background characteristics, 2006/2007 Nairobi Slum HIV Prevalence Survey (NSHPS) and 2008/2009 Kenya Demographic and Health Survey (KDHS).

	Nairobi slums (NSHPS)		Nairobi non-slum (KDHS)		Other slums (KDHS)		Non-slum other urban (KDHS)		Rural (KDHS)	
	%	*N*	%	*N*	%	*N*	%	*N*	%	*N*
**Total**	**29.1**	**2742**	**6.0**	**584**	**3.2**	**306**	**8.2**	**1076**	**53.5**	**5048**
**Age groups**										
15–19	15.2	413	11.4	66	14.4	44	15.3	165	25.5	1288
20–24	23.4	636	24.3	142	21.2	65	20.9	224	18.8	947
25–29	20.7	564	23.9	139	21.9	67	22.0	236	14.2	717
30–34	15.0	409	15.8	92	15.7	48	15.8	170	13.6	687
35–39	11.5	314	11.1	65	8.2	25	10.1	109	9.4	477
40–44	7.5	205	8.1	48	13.4	41	10	107	9.5	481
45+	6.6	180	5.5	32	[5.2]	16	5.9	63	8.9	450

**Sex**										
Female	63	1713	49	287	58.5	179	53.4	575	55.1	2779
Male	37	1008	51	297	41.5	127	46.6	501	44.9	2268

**Age at first sex**										
<15 years	14.8	403	8.7	51	13.7	42	10.1	108	17.1	861
15–19 years	53.4	1452	55.9	327	47.7	146	51.1	549	48.2	2435
20+ years	14.5	394	22.0	129	23.9	73	21.6	232	12.5	633
Never had sex	12.8	348	10.7	63	9.8	30	13.4	144	18.2	918
Inconsistent/refused	4.6	124	2.6	15	[4.9]	15	3.8	41	4.0	201

**Current marital status**										
Never married	27.1	737	42.6	249	24.5	75	30.2	324	40.2	2028
Currently in union	62.4	1697	53.1	310	59.5	182	61.4	660	52.0	2626
Formerly married	10.5	287	4.4	25	16.0	49	8.4	91	7.8	393

**Highest education**										
None	3.8	103	1.9	11	9.5	29	4.1	45	7.2	362
Primary	64.7	1761	25.0	146	54.9	168	38.9	418	62.6	3111
Secondray or higher	31.5	857	73.1	427	35.6	109	57	612	31.2	1575

**Ethnicity**										
Kikuyu	30.4	827	33.3	194	11.8	36	24.3	262	15.7	790
Kamba	19.0	517	14.1	82	[3.9]	12	7.5	81	11.6	587
Luhya	16.5	449	17.5	102	24.6	75	17.8	192	15.9	802
Luo	19.1	521	15.3	90	13.4	41	10.7	115	13.1	663
Others	15.0	407	19.9	116	46.2	141	39.6	426	43.7	2204

[ ] Based on fewer than 25 cases.

**Table 2:** Percentage respondents HIV positive by selected characteristics and place of residence. Nairobi Slum HIV Prevalence Survey (NSHPS), 2006/2007, Kenya Demographic and Health Survey (KDHS), 2008/2009.

	**Slum areas (NSHPS)**				**Non-slum urban (KDHS)**				**Rural (KDHS)**			
**Characteristic**	**Men**	**Women**	**All**	***n***	**Men**	**Women**	**All**	***n***	**Men**	**Women**	**All**	***n***
**Total**	9.5	13.2	11.8	3048	2.9	7.7	5.3	1332	4.5	7.2	6	5048
**Age group**												
15–19	2.7	6.0	5.0	463	1.0	0.0	0.6	66	0.6	3.0	1.8	1288
20–24	2.9	8.4	7.2	705	0.8	6.0	3.4	142	1.8	6.5	4.3	948
25–29	6.5	15.1	12	635	4.3	5.4	4.9	139	8.3	11.4	10.2	717
30–34	10.5	17.2	14.2	459	4.5	18.6	11.3	92	6.8	8.4	7.7	687
35–39	17.1	18.5	17.8	342	5.3	5.6	4.8	65	11.7	8.9	10.1	477
40–44	10.6	23.6	18.1	248	1.7	14.8	8.8	48	6.0	9.8	8.1	481
45+	20.0	20.5	20.2	198	2.1	6.9	3.9	32	4.9	6.0	5.6	450
**Ethnicity**												
Kikuyu	4.7	10.2	8.3	875	0.6	4.9	2.9	407	2.4	5.5	4.2	790
Kamba	6.8	10.5	8.8	533	3.8	8.6	6.1	148	1.9	4.3	3.2	588
Luhya	10.3	15.8	13.7	524	0.0	8.5	3.7	218	2.1	9.7	6.1	802
Luo	23.6	21.8	22.4	566	9.2	20.5	15.1	159	19	23.5	21.4	664
Others	5.4	8.7	7.5	549	4.0	5.0	4.5	400	2.2	3.0	2.7	2204
**Current marital status**												
Never married/in union	3.0	6.4	5.0	819	1.1	3.9	2.4	490	1.5	3.7	2.5	2028
Currently in union	11.3	11.7	11.5	1889	4.2	7.4	5.8	778	6.6	6.0	6.2	2627
Formerly in union	19.0	33.1	29.8	339	[0.0]	27.1	21.9	64	16.4	24.7	22.4	393
**Age at first sex**												
<15 years	7.2	24.2	15.4	449	2.3	25	7.9	114	2.8	14.4	7.8	861
15–19 years	11.1	14.1	13.0	1611	3.4	8.1	5.6	718	5.2	8.0	6.8	2435
20+ years	9.8	12.8	11.5	468	4.5	5.1	4.9	287	10.1	3.6	6.8	633
Never had sex	3.3	4.1	3.9	381	0.0	1.1	0.6	174	0.5	1.2	1	918
Inconsistent/refused	13.3	7.4	9.4	139	[0.0]	18.4	17.1	41	[0.0]	9.1	9.0	201
**Education**												
None	[20.8]	20.4	20.5	132	[0.0]	[5.3]	3.8	26.0	5.7	5.1	5.5	362
Primary	9.8	14.1	12.7	1942	3.9	8.6	6.8	384	4.8	8.0	6.6	3110
Secondary+	8.6	9.3	8.9	974	2.8	7.1	4.8	923	3.6	6.3	4.9	1575
**Wealth quintiles**												
Poorest	9.4	14.5	12.2	565	1.8	5.0	3.1	96	1.8	5.7	4.3	728
Second	9.3	19.5	15.0	659	7.5	9.9	8.7	161	3.6	7.2	5.7	1033
Middle	7.8	13.3	11.2	596	3.7	7.7	5.7	368	5.4	9.2	7.4	1047
Fourth	11.7	11.1	11.3	594	1.0	6.3	3.7	403	4.2	3.8	4	1080
Least poor	9.9	8.8	9.2	633	2.1	8.6	5.6	304	6.0	9.7	7.9	1159
**Circumcised men**												
No/Don't know	20.8			216	3.3			60	14.0			365
Yes	6.9			929	2.8			601	2.6			1903

[ ] based on fewer than 25 cases.

**Table 3:** Odds ratios of being HIV positive by place of residence and other background characteristics, 2006/2007 Nairobi Slum HIV Prevalence Survey and 2008/2009 Kenya Demographic and Health Survey.

**Place of residence**	**Men**			**Women**		
	**Unadjusted odds ratios**	**95% CI**	***n***	**Unadjusted odds ratios**	**95% CI**	***n***
All slums	1.0		1185	1.0		1986
Other urban	0.39	(0.26, 0.59)	712	0.61	(0.46, 0.80)	820
Rural	0.44	(0.33, 0.58)	2018	0.52	(0.43, 0.64)	2718

	**Adjusted odds ratios**	**95% CI**	***n***	**Adjusted odds ratios**	**95% CI**	***n***
**Place of residence**						
All slums	1.0		1185	1.0		1986
Other urban	0.51	(0.32, 0.81)	712	0.63	(0.33, 1.24)	820
Rural	0.61	(0.44, 0.84)	2018	0.51	(0.30, 0.86)	2718
**Age group**						
15–19	1.0		832	1.0		1083
20–24	1.39	(0.59, 3.25)	699	1.25	(0.82, 1.89)	1292
25–29	3.11	(1.34, 7.18)	628	2.48	(1.62,3.80)	996
30–34	4.60	(1.94, 10.89)	612	2.7	(1.72, 4.24)	741
35–39	7.54	(3.14, 18.13)	456	2.34	(1.47, 3.74)	584
40–44	4.66	(1.84, 11.77)	349	2.26	(1.38, 3.72)	444
45+	6.03	(2.42,15.00)	339	1.49	(0.86, 2.55)	384
**Ethnicity**						
Kikuyu	1.0		744	1		1222
Kamba	1.3	(0.71, 2.27)	482	1.22	(0.75, 1.97)	571
Luhya	1.43	(0.83, 2.46)	659	1.38	(0.89, 2.14)	872
Luo	7.88	(4.97, 12.51)	592	3.05	(2.07, 4.49)	865
Others	1.18	(0.70, 1.97)	1438	1.08	(0.68, 1.73)	1994
**Current marital status**						
Never married/in union	1.0		1641	1.0		1587
Currently in union	1.36	(0.79, 2.34)	2096	0.88	(0.64, 1.21)	3336
Formerly in union	3.56	(1.81, 6.96)	178	3.78	(2.62, 5.43)	602
<15 years	1.0		801	1.0		676
15–19 years	1.34	(0.91, 1.96)	1881	0.56	(0.43,0.72)	2811
20+ years	1.36	(0.85, 2.18)	620	0.38	(0.26, 0.56)	804
Never had sex	0.58	(0.29, 2.00)	558	0.18	(0.10, 0.32)	899
Inconsistent/refused	0.03	(1.09, 7.23)	55	0.43	(0.27, 0.68)	334
**Education**						
None	1.0		164	1.0		601
Primary or lower	1.3	(0.62, 2.89)	2103	0.90	(0.61, 1.33)	3207
Secondary	0.89	(0.40, 1.95)	1648	0.83	(0.54, 1.27)	1716
**Wealth quintiles**						
Poorest	1.0		712	1.0		1022
Second	1.02	(0.64, 1.61)	819	1.13	(0.83, 1.55)	1042
Middle	1.32	(0.83, 2.09)	820	1.12	(0.82, 1.55)	1110
Fourth	0.89	(0.54, 1.48)	765	0.84	(0.60, 1.17)	1172
Least poor	1.26	(0.78,2.04)	799	0.95	(0.67, 1.33)	1178
**Interactions**						
Ethnicity by place	Not significant					
Kamba, other urban				1.44	(0.50, 4.16)	
Kamba, rural				0.90	(0.36, 2.27)	
Luhya, other urban				1.49	(0.56, 3.94)	
Luhya, rural				1.26	(0.61, 2.60)	
Luo, other urban				1.61	(0.68, 3.81)	
Luo, rural				1.96	(1.03, 3.74)	
Others, other urban				0.72	(0.28, 1.86)	
Others, rural				0.55	(0.27, 0.12)	
